# A study on surprisal and semantic relatedness for eye-tracking data prediction

**DOI:** 10.3389/fpsyg.2023.1112365

**Published:** 2023-02-02

**Authors:** Lavinia Salicchi, Emmanuele Chersoni, Alessandro Lenci

**Affiliations:** ^1^Department of Chinese and Bilingual Studies, The Hong Kong Polytechnic University, Kowloon, Hong Kong SAR, China; ^2^Computational Linguistics Laboratory (CoLing Lab), University of Pisa, Pisa, Italy

**Keywords:** cognitive modeling, surprisal, semantic relatedness, cosine similarity, language models, distributional semantics, eye-tracking

## Abstract

Previous research in computational linguistics dedicated a lot of effort to using language modeling and/or distributional semantic models to predict metrics extracted from eye-tracking data. However, it is not clear whether the two components have a distinct contribution, with recent studies claiming that surprisal scores estimated with large-scale, deep learning-based language models subsume the semantic relatedness component. In our study, we propose a regression experiment for estimating different eye-tracking metrics on two English corpora, contrasting the quality of the predictions with and without the surprisal and the relatedness components. Different types of relatedness scores derived from both static and contextual models have also been tested. Our results suggest that both components play a role in the prediction, with semantic relatedness surprisingly contributing also to the prediction of function words. Moreover, they show that when the metric is computed with the contextual embeddings of the BERT model, it is able to explain a higher amount of variance.

## 1. Introduction

Eye-tracking data recorded during reading provide important evidence about the factors influencing language comprehension (Rayner et al., 1989; Rayner, 1998). In the investigation of potential predictors of human reading patterns, cognitive studies have focused their attention on two specific factors, among the others: (i) the semantic coherence of a word with the rest of the sentence (Ehrlich and Rayner, 1981; Pynte et al., 2008; Mitchell et al., 2010), which is typically assessed *via semantic relatedness* metrics (usually the *cosine*) computed with *distributional word embeddings*, and (ii) the predictability of the word from its previous context, as measured by *surprisal* (Hale, 2001; Levy, 2008). Initially, the two factors were considered separately, and the general idea was that words having low semantic coherence and low in-context predictability (i.e., high surprisal) induce longer reading times. This hypothesis was instead questioned by Frank (2017), who argued that previous findings had to be attributed to a confound between semantic relatedness and word predictability and that the effect of the former disappeared once surprisal was factored out.

Our work aims at providing further evidence about the complex interplay between semantic relatedness and surprisal as predictors of eye-tracking data. For example, it is unclear whether the fact that no independent effect of relatedness has been found depends on the specific word embedding model being used for measuring it. In fact, there is a large variety of Distributional Semantic Models (DSMs) that are trained with different objectives, and they have been shown to perform differently depending on the task (Lenci et al., 2022). Moreover, the recent introduction of contextual embedding models such as ELMo (Peters et al., 2018) and BERT (Devlin et al., 2019) has also radically changed the way semantic relatedness can be assessed. In particular, contextual embeddings now make it possible to compare the semantic representations of *words in specific contexts* (*token-level representations*), and not just type-level representations that tend to conflate multiple senses of the same word.

The goals of this paper can thus be summarized as follows:

1. Investigating whether distributional measures of semantic relatedness between a word and its previous contexts are indeed made redundant by surprisal, or have instead an autonomous explanatory role to model eye-tracking data;
2. Looking into different types of word embeddings, to check whether “classical” static models and contextual ones interact differently or not with surprisal.

To explore these issues, we implemented four different linear models to predict three eye-tracking features on two eye-tracking corpora: i) a baseline with word-level features, ii) a model with baseline features and the surprisal between target word and context, iii) a model with baseline features and the relatedness between the vector representing the target word and the vector representing the context, and iv) a model with all the above-mentioned regression features. While surprisal has been consistently computed using a state-of-the-art neural language model GPT2-xl (Radford et al., 2019), the vectors employed in the cosine similarity calculation were obtained using either SGNS (Mikolov et al., 2013) or BERT (Devlin et al., 2019), to compare static and contextual word embedding models.

Our results show that the models including both relatedness and surprisal perform better than the other three, suggesting that, despite the overlap between the two, they contribute differently in explaining the variance in the data. Furthermore, when comparing the models using only relatedness, we noticed that BERT vectors outperform SGNS ones, confirming the added value of contextual embeddings when modeling the relatedness of words in contexts. Finally, we investigated how our models predict eye-tracking feature values for different parts of speech, and we found that while surprisal helps on content words, semantic relatedness contributes to improving the predictions on both function and content words.

## 2. Computational models of human reading times: Surprisal and semantic relatedness

Since the cognitive processes of meaning construction involve the integration of individual word meanings into the syntactic and semantic context, the literature in natural language processing and cognitive science got interested in how such contextual effects on word fixations could be modeled. A first class of computational models has relied on distributional semantics to assess the relatedness of a word with its wider semantic context (Section 2.1); another class of models has explored the connection between the logarithmic probabilities of words in context and their processing difficulty (Section 2.2).

### 2.1. Computational measures for semantic coherence

A fruitful line of research has been investigating the usage of cosine similarity between word embeddings for predicting reading times. The employment of word vectors for modeling reading times originated from classical DSMs (Lenci and Sahlgren, 2023). Pynte et al. (2008) and Mitchell et al. (2010) used the semantic distance between a target word and the context as a predictor, measured as 1 min the traditional cosine similarity metric (Turney and Pantel, 2010; Lenci, 2018). The context was in turn modeled as the sum of the distributional vectors representing the words before the target. These studies found strong correlations between semantic distance and reading times: The more semantically related the words, the shorter the fixation durations.

Originally, vector spaces were obtained from the extraction and counting (hence the name of *count models*) of the co-occurrences between the target words and the relevant linguistic contexts. Raw co-occurrences were usually weighted *via* different types of statistical association measures [e.g., Mutual Information, log-likelihood; see Evert (2005) for an overview] and then the vector space was optionally transformed with some algebraic operation for dimensionality reduction, such as Singular Value Decomposition (Landauer and Dumais, 1997; Bullinaria and Levy, 2012). The contexts could consist either in the words occurring within a window surrounding the target (Lund and Burgess, 1996; Sahlgren, 2008), or in the words linked to the target by syntactic (Padó and Lapata, 2007; Baroni and Lenci, 2010) or semantic relations (Sayeed et al., 2015).

Later, with the increasing success of deep learning techniques in Natural Language Processing, the so-called *predict models* established themselves as a new standard (Mikolov et al., 2013; Bojanowski et al., 2017). In such models, the learning of word vectors is based on neural network training and framed as a self-supervised language modeling task. One of the most popular predict DSMs is Word2Vec (Mikolov et al., 2013), which includes two main architectures: CBOW, trained for predicting a target word given the context surrounding it, and Skip-Gram, whose learning objective is to predict the surrounding context given a target word. The most common implementation of Skip-Gram makes use of negative sampling (SGNS), whose objective is to discriminate between word sequences that are actually occurring in the data (positive samples) and "corrupted" samples, which are obtained by randomly replacing a word in a true sequence from the corpus (negative samples).

One of the main limitations of “traditional” word embeddings, both count and predict ones, is that they provide *static* representations of the semantics of a word. They assign a single embedding to each word type, thereby conflating the possible senses of a lexeme and hampering the possibility to address the pervasive phenomena of polysemy and homography. For example, *bank* as a financial agency will have the same vector representation of *bank* as the bank of the river. This way, lexical semantic representations are built at the *type* level only, and the embedding will be a sort of distributional summary of all the instances of a word, no matter how different their senses might be (and probably, the most frequent senses would obscure the minority ones).

The most recent generation of DSMs is said to be *contextual* because they produce a distinct vector for each word instance in context, that is a *token* level representation (Peters et al., 2018; Devlin et al., 2019; Liu et al., 2019). Contextual DSMs generally rely on a multi-encoder network and the word vectors are learned as a function of the internal states, so that a word appearing in different sentence contexts determines different activation states and, as a consequence, is represented by a different vector.

Most contextual DSMs are based on *Transformers* (Vaswani et al., 2017), which use a self-attention mechanism (Bahdanau et al., 2014) for getting the most salient elements in a sentence context and assign them higher weights. BERT (Devlin et al., 2019) is probably the most popular model for generating contextual word representations. BERT is trained on a masked language modeling objective function: random words in the input sentences are replaced by a ‘[MASK]' token and the model attempts to predict the masked word based on the surrounding context. Simultaneously, BERT is optimized on a next sentence prediction task, as the model receives sentence pairs in input and has to predict whether the second sentence is subsequent to the first one in the training data. It should be noticed that BERT is defined as *deeply bidirectional* as, in fact, it takes into account the left-hand and the right-hand context of a word to predict the word filling the masked token. The contextual embeddings produced by BERT have been shown to improve the state-of-the-art performance in several Natural Language Processing tasks (Devlin et al., 2019) and it has been reported that its multilingual versions (i.e., Multilingual BERT, XLM) are able to predict human fixations in multiple languages (Hollenstein et al., 2021, 2022a,b). Significantly, it was shown that it is possible to extract semantic representations at the type level from BERT just by averaging token vectors of randomly-sampled sentences, and those can achieve a performance close to traditional word embeddings on word similarity tasks (Bommasani et al., 2020; Chronis and Erk, 2020; Lenci et al., 2022) and on word association modeling (Rodriguez and Merlo, 2020).

### 2.2. Computational measures for word predictability

A significant part of the psycholinguistic and computational studies modeled naturalistic reading data by means of language model probabilities, being inspired by *surprisal theory* (Hale, 2001, 2016), with the idea that the predictability of a word is the main factor determining the reading times. More specifically, the processing difficulty of a word is considered to be proportional to its *surprisal*, that is, the negative logarithm of the probability of the word given the context. Several studies based on language models adopted surprisal theory as a reference framework for the prediction of eye-tracking data (Demberg and Keller, 2008; Frank and Bod, 2011; Fossum and Levy, 2012; Monsalve et al., 2012; Smith and Levy, 2013). The predictions were typically evaluated on the Dundee Corpus (Kennedy et al., 2003), as one of the earliest corpora with gold standard annotations of eye-tracking measures.

Later research has focused on the quality of the language model to estimate conditional probabilities, finding that models with lower perplexity are a better fit to human reading times (Goodkind and Bicknell, 2018). Following studies confirmed the model perplexity as a significant determinant, making use of more and more advanced neural architectures, such as LSTM (van Schijndel and Linzen, 2018), GRU (Aurnhammer and Frank, 2019), Transformers (Merkx and Frank, 2021), GPT-2 (Wilcox et al., 2020).

Is contextual predictability, that is surprisal, all we need to model human reading behavior? Some recent results suggest that this may not be the case. Goodkind and Bicknell (2021), for example, investigated the role played on local word statistics, such as word bigram and trigram probability, in sentence processing, and consequently their impact on reading times, finding that they affect processing independently of surprisal. Moreover, Hofmann et al. (2021) compared different models for computing surprisal as predictors of eye-tracking fixations and found that they explain different and independent proportions of variance in the viewing parameters. For example, classical n-gram-based language models are better at predicting metrics related to short-range access, while RNN models better predict the early preprocessing of the next word.

The models of the GPT family are based on Transformer architectures (Radford et al., 2018, 2019; Brown et al., 2020). Differently from BERT, GPT is a uni-directional, autoregressive Transformer language model, which means that the training objective is to predict the next word, given all of the previous words. GPT-2, in particular, has been commonly used in eye-tracking studies, as the surprisal scores computed by this language model have been proved to be strong predictors of reading times and eye fixations in English (Hao et al., 2020; Wilcox et al., 2020; Merkx and Frank, 2021) and in other languages (e.g., Dutch, German, Hindi, Chinese, Russian) (Salicchi et al., 2022).

The research work on semantic relatedness and surprisal led Frank (2017) to ask whether these two factors have actually independent effects in the modeling of reading times. The question was motivated by the fact that not all the studies on reading times found effects associated with semantic relatedness (e.g., Traxler et al., 2000; Gordon et al., 2006), although vector space metrics clearly proved to be useful for modeling other types of experimental data on naturalistic reading, such as the N400 amplitude in EEG recordings (Frank and Willems, 2017). Frank suggested that, since DSMs like Word2Vec (Mikolov et al., 2013) are based on word co-occurrence and are optimized for predicting words in context, previous results were due to a confound between semantic relatedness and word predictability. Indeed, when surprisal was factored out, the author showed that the semantic distance effects disappeared. Moreover, the different results obtained in modeling the N400 component in the EEG data were attributed to differences in the stimuli presentation method: while in eye-tracking participants read the text naturally, in many EEG studies words are presented one at a time with unnaturally long durations. Following the findings of Wlotko and Federmeier (2015) and Frank (2017) pointed out that, the more natural the presentation rates of the words in the experimental setting in EEG, the smaller the semantic relatedness effects on N400 data tend to be, with no effects at all for behavioral metrics on naturalistic reading. Is distributional semantic relatedness really made redundant by surprisal, or were the results by Frank (2017) also conditioned by the specific type of embeddings used in the experiments? The analyzes in Sections 3, 4 aim at clarifying this issue.

## 3. Materials and methods

### 3.1. Definition of eye-tracking metrics in psycholinguistic studies

Several metrics have been defined to describe eye movement features (Rayner, 1998). In this work, we focus on first fixation duration, number of fixations and total reading time. The first fixation duration (FFD), that is the time spent fixing a word for the first time, is typically associated with lexical information processing, like lexical access (Inhoff, 1984), which is heavily affected by word frequency (Balota and Chumbley, 1984). Fast word recognition is obtained when a word can be recognized with a single glance. In this sense, a short FFD reflects a quick and successful lexical access (Hofmann et al., 2021).

However, several words may not be accessed immediately. Words may receive multiple fixations before the eyes move to the next word, and this is reflected by the number of fixations (NF), depending on the integration of the word within the sentence semantics or syntax (Frazier and Rayner, 1982). An alternative metric for this “delayed” lexical access is known as *gaze duration*, which computes directly the sum of the duration of individual fixations before moving to the next word (Inhoff and Radach, 1998; Rayner, 1998).

Finally, the total reading time (TRT), as the sum of all fixation durations on the word, including regressions, is affected by both lexical and sentence-level processing. The TRT is likely to indicate the time required for the full semantic integration of the word in the sentence context (Radach and Kennedy, 2013).

What are the factors affecting word fixations during reading? There is a general consensus that word position, word length, and the number of syllables within the word affect language processing and, consequently, reading behavior and fixations (Just and Carpenter, 1980). It has also been observed that low-frequency words tend to have longer gaze durations and, additionally, they lead to longer gaze on the immediately following words, a phenomenon typically referred to as *spillover effect* (Rayner and Duffy, 1986; Rayner et al., 1989; Remington et al., 2018). A common explanation is that rare and longer words have a higher cognitive load, as they require more time for the semantic integration in the sentence context (Pollatsek et al., 2008), and therefore they may influence the processing of the following words.

### 3.2. Eye-tracking corpora

Traditional corpora annotated with eye-tracking data consist of short isolated sentences (or even single words) with particular structures or lexemes, in order to investigate specific syntactic and semantic phenomena. In the present work, we use GECO (Cop et al., 2017) and Provo (Luke and Christianson, 2018), two eye-tracking corpora containing long, complete, and coherent texts.

**GECO** is a bilingual corpus in English and Dutch composed of the entire Agatha Christie's novel *The Mysterious Affair at Styles*. The corpus is freely downloadable with a related dataset containing eye-tracking data of 33 subjects (19 of them bilingual, 14 English monolingual) reading the full novel text, presented paragraph-by-paragraph on a screen^1^. In total, GECO is composed of 54,364 tokens.

**Provo** contains 55 short English texts about various topics, with 2.5 sentences and 50 words on average, for a total of 2, 689 tokens, and a vocabulary of 1,197 words. These texts were read by 84 native English speakers and their eye-tracking measures were collected and made publicly available online^2^.

GECO and Provo are particularly interesting for our goals because they are corpora of naturalistic reading since data have been recorded from subjects reading real texts, instead of short stimuli created *in vitro*. For every word in the corpora, we extracted the mean total reading time, mean first fixation duration, and mean number of fixations. Mean values were obtained by averaging over the subjects. The choice of modeling mean eye-tracking measures is justified by the high inter-subject consistency of the recorded data.

### 3.3. Method

We implemented and compared four main types of linear models (see Table 1):

1. A baseline model with word-related statistics that are known to influence sentence and word processing (i.e., word frequency, word length, word position within the sentence, previous word frequency, previous word length, and whether or not the previous word was fixated);
2. Two models combining baseline features and cosine similarity, one using Skip-Gram vectors (SGNS), one using BERT vectors;
3. One model with baseline features + surprisal computed using GPT2-xl;
4. Two models with baseline features + surprisal computed using GPT2-xl + cosine similarity, one using SGNS vectors, one using BERT vectors.

**Table 1:** Summary of the linear models implemented for the experiments.

**Model name**	**Features**
BL	Word frequency Word length Word position within the sentence Previous word frequency Previous word length Whether or not the previous word was fixated
BL-cos	Baseline features (same as BL) Cosine similarity (BERT vectors)
	Baseline features (same as BL) Cosine similarity (SGNS vectors)
BL-sur	Baseline features (same as BL) Surprisal (GPT2-xl)
BL-sur-cos	Baseline features (same as BL) Surprisal (GPT2-xl) Cosine similarity (SGNS vectors)
	Baseline features (same as BL) Surprisal (GPT2-xl) Cosine similarity (BERT vectors)

Recent works have cast doubts on the application of cosine in similarity task while employing contextual vector models. In fact, in contextual embeddings a small number of dimensions (e.g., 3-5) tend to dominate the similarity metric, accounting for most of the data variance (Timkey and van Schijndel, 2021). Moreover, it has been shown that the removal of the outlier dimensions leads to drastic performance drops both in language modeling and in downstream tasks (Kovaleva et al., 2021).

To address this issue, for similarity tasks it has been suggested to correct the comparisons by discounting the “rogue” dimensions or to adopt metrics based on the rank of the dimensions themselves, rather than on their absolute values (Timkey and van Schijndel, 2021). In order to take into account the potential effect of rogue dimensions on computing cosine similarity with BERT, we followed the latter suggestion and we also implemented two further models, in which we use Spearman correlation instead of cosine similarity.

Rank-based metrics have been reported to outperform vector cosine in semantic relatedness tasks (Santus et al., 2016a,b, 2018; Zhelezniak et al., 2019), and it has been shown that Spearman itself is more correlated with human judgments than cosine (Timkey and van Schijndel, 2021). For each of the resulting eight models, the values to be predicted were first fixation duration (FFD), number of fixations (NF) and total reading time (TRT). We predicted those metrics on both GECO and Provo corpus. We also experimented with models with and without interactions between the features. The models were implemented using the generalized linear models available in R, which have also been used for the statistical analysis.

After we fitted the data of the eye-tracking features with each model, we compared them using the corrected Akaike Information Criterion (AICc) in order to determine the extent to which the goodness of fit improves with the addition of semantic relatedness and surprisal as predictors. Additionally, we also analyzed i) the correlations between linear model errors (as Mean Absolute Error, MAE) and word features, and ii) which parts of speech are easier or harder for each model to predict.

### 3.4. Regression features

#### 3.4.1. Baseline features

The baseline model includes the following word features: i) the target word and previous word length, computed as the number of letters within the word to be predicted; ii) the target word and previous word frequency, whose values are extracted from Wikipedia;^3^ iii) the target word position, as the index of the word within the current sentence; iv) a Boolean value corresponding to 1 if the word preceding the target word was fixated, 0 otherwise. The baseline features are the same used by Frank (2017).

#### 3.4.2. Metrics of semantic relatedness

To compute the semantic relatedness between the context and the target word, we extracted vectors for each word, represented the sentence context with a vector, and finally computed, alternatively, the *cosine similarity* or the *Spearman correlation* between the context and the target vectors (the latter metric was used only with the BERT vectors only).

With **SGNS** embeddings, we extracted the pre-trained vectors for each word, and we computed the context vector using an additive model: We summed the vectors of all the words preceding the target and took this as the context representation. For example, given the sentence *The dog chases the cat*, if the target word is *chases*, the context vector will be $\vec{The}+\vec{dog}$, while if the target word is *cat*, the context vector will be $\vec{The}+\vec{dog}+\vec{chases}+\vec{the}$.

On the other hand, given the bidirectional nature of the **BERT** language model, the input to extract the embeddings from this model required a special preprocessing, since we wanted to avoid the model to “see the future,” by having the target word vector including information also from the right-hand context. Therefore, we fed BERT with sub-sentences. For instance, given the sentence *The dog chases the cat*, we generated the following sub-sentences:

S[0] = [*The*]
S[1] = [*The dog*]
S[2] = [*The dog chases*]
S[3] = [*The dog chases the*]
S[4] = [*The dog chases the cat*]

For each target word, we extracted its vector, when the lexeme occurs at the end of a sub-sentence (e.g., *The* will be extracted in S[0], *dog* in S[1], *chases* in S[2], and so on).

Regarding the context, we used the vector of the special token [CLS], which is created by BERT as a global representation of the input sentence, taking into account how salient each word is for the sentence's meaning. Again, to avoid a representation of the target word itself within the [CLS] vector, we computed the cosine similarity and the Spearman correlation between the target word embedding, and the [CLS] vector of the previous sub-sentence. For example, if *cat* is the target word, we computed the cosine similarity between $\vec{cat}$ from S[4] and $\vec{CLS_{S\left[3\right]}}$. In order to find the optimal layer for the computation of the similarity scores, we extracted vectors from all the 24 layers of BERT Large and computed the Spearman correlations with each one of the target features.

**Figure 1:**
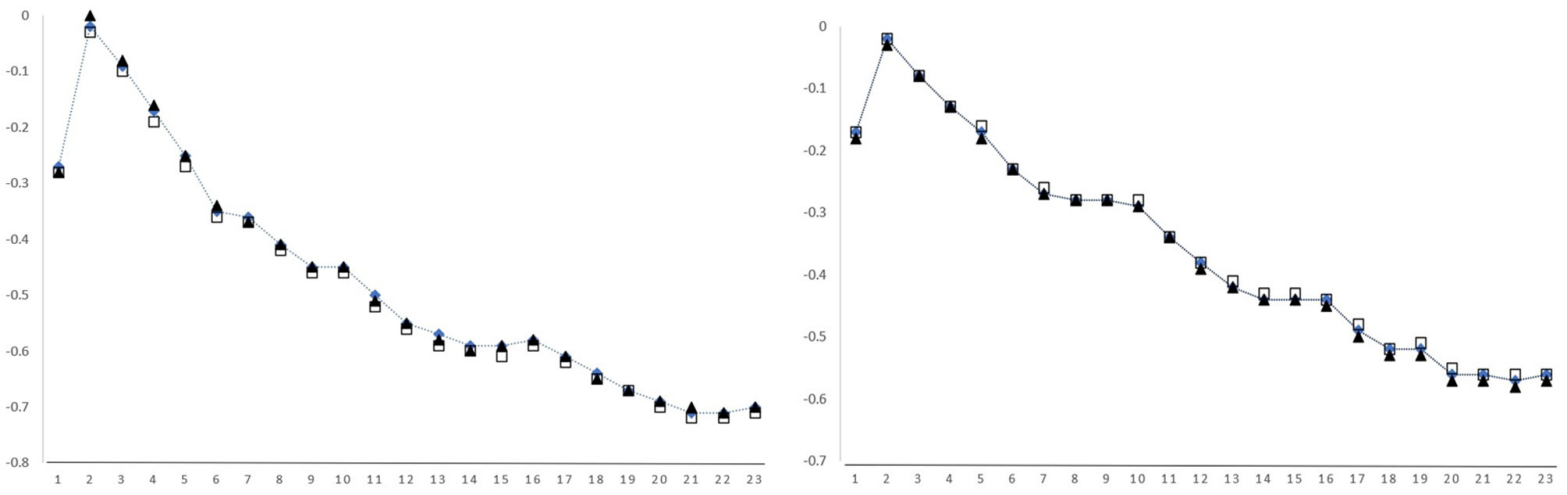
Spearman correlations between TRT (dot line), FFD (square), and NF (triangle) and the cosine similarity using vectors produced by different layers of BERT Large, on GECO **(left)** and Provo **(right**). Layer 24, whose values are systematically higher than the average, is intentionally left out for plot reading purposes.

The results can be seen in Figure 1. Consistently with the findings of Salicchi et al. (2021), the layers with the highest absolute correlation values are the ones immediately before the last one. We chose layer 22 as the one with the highest inverse correlation to our data.

#### 3.4.3. Surprisal

To model the influence of word predictability on eye-tracking measures, we included in the regression models the surprisal of the target words given their previous context. For each target word we computed the surprisal as the negative logarithm of its probability given all the words preceding the target:


(1) $$\begin{array}{l} surprisal\left(w_{n}\right)=-\log P\left(w_{n}|w_{0},w_{1},...,w_{n-1}\right) \end{array}$$

The probability *P* is computed by GPT2-xl, the largest publicly available version of GPT-2. Similarly to the original model, GPT2-xl was also trained on the WebText corpus (40 GB of text data), but it has a larger architecture (48 layers, for a total of 1542M parameters) and was shown to have the lowest perplexity on the evaluation corpora of Radford et al. (2019).

## 4. Results and discussion

### 4.1. General analysis

#### 4.1.1. Cosine similarity vs. Spearman correlation

We first checked whether Spearman correlation was a better similarity metric than cosine with BERT contextual embeddings. Therefore, we compared BL-cos and BL-Spearman, namely models with baseline features and the similarity metric only, and we compared BL-sur-cos and BL-sur-Spearman, which are the models using baseline features, surprisal, and the similarity metric. The AICc values reported in Tables 2–5 clearly show that cosine similarity is a better predictor of eye-tracking features than Spearman correlation: on GECO, the difference between BL-cos and BL-Spearman is 1,279, and between BL-sur-cos and BL-sur-Spearman is 901; on Provo the differences are 333 and 318, respectively. Given these results, we henceforth focus our analyzes only on cosine similarity and its relationship with surprisal. Our findings suggest that, within the linear models we propose, BERT embeddings anisotropy does not affect the eye movements modeling, and therefore, cosine similarity is a suitable feature to be used for this eye tracking feature prediction task.

**Table 2:** Average AICc, and AICc for TRT, FFD, and NF on GECO with SGNS vectors.

	**Avg**		**TRT**		**FFD**		**NF**	
**Model**	**AICc**	**Delta**	**AICc**	**Delta**	**AICc**	**Delta**	**AICc**	**Delta**
BL-sur-cos	60,286	0	88,611	0	80,296	0	11,951	0
BL-sur	60,492	206	88,835	224	80,576	280	12,065	115
BL-cos	60,982	696	89,409	798	80,903	607	12,634	683
BL	61,466	1,180	89,948	1,337	81,483	1,186	12,969	1,018

**Table 3:** Average AICc, and AICc for TRT, FFD, and NF on GECO with BERT vectors.

	**Avg**		**TRT**		**FFD**		**NF**	
**Model**	**AICc**	**Delta**	**AICc**	**Delta**	**AICc**	**Delta**	**AICc**	**Delta**
BL-sur-cos	59,566	0	87,758	0	79,232	0	11,709	0
BL-cos	60,151	585	88,413	654	79,697	465	12,346	637
BL-sur-Spearman	60,467	901	88,803	1,045	80,538	1,307	12,060	350
BL-sur	60,492	926	88,835	1,077	80,576	1,345	12,065	356
BL-Spearman	61,430	1,864	89,902	2,145	81,432	2,200	12,957	1,247
BL	61,466	1,900	89,948	2,190	81,483	2,251	12,969	1,259

**Table 4:** Average AICc, and AICc for TRT, FFD, and NF on Provo with SGNS vectors.

	**Avg**		**TRT**		**FFD**		**NF**	
**Model**	**AICc**	**Delta**	**AICc**	**Delta**	**AICc**	**Delta**	**AICc**	**Delta**
BL-sur-cos	279	0	1,309	0	288	0	–762	0
BL-sur	391	112	1,436	127	441	153	–704	58
BL-cos	437	158	1,468	159	406	118	–594	168
BL	619	340	1,683	374	643	354	–470	292

**Table 5:** Average AICc, and AICc for TRT, FFD, and NF on Provo with BERT vectors.

	**Avg**		**TRT**		**FFD**		**NF**	
**Model**	**AICc**	**Delta**	**AICc**	**Delta**	**AICc**	**Delta**	**AICc**	**Delta**
BL-sur-cos	67	0	1,081	0	–88	0	–791	0
BL-cos	196	129	1,216	135	–0.26	87	–627	165
BL-sur-Spearman	385	318	1,429	348	434	521	–707	85
BL-sur	391	324	1,436	355	441	529	–704	88
BL-Spearman	529	462	1,674	593	633	721	–474	315
BL	619	552	1,683	602	643	730	–470	321

#### 4.1.2. Linear models comparison

For each implemented model, we used AICc values to determine which one was the best fit for the data. On both corpora, we notice that the best predictor of eye-tracking features is BL-sur-cos, including the interactions between baseline features, but with no interactions between cosine and surprisal. The fact that the regression model using both surprisal and cosine consistently performs better than the ones using only one of the two is strong evidence that they are both explanatory factors of reading times. Furthermore, while comparing BL-cos-sur with SGNS embeddings, and BL-cos-sur with BERT embeddings, it is possible to notice how the usage of the latter set of vectors improves the model (AICc values on GECO: 60,286 with SGNS-59,566 with BERT; AICc values on Provo: 279 with SGNS-67 with BERT).

Looking at the *p*-values of the regression features of our BL-sur-cos model, we observe that both cosine similarity and surprisal are statistically highly significant at *p* < 0.001 (for a complete analysis of regression features significance scores see Appendix 1). Although the combination of both cosine similarity and surprisal is the best performing model on both corpora, it is useful to focus also on the performances of BL-cos, and BL-sur while employing different vector models for BL-cos, to get further insights on the different contributions of surprisal and cosine similarity. We performed nested model comparisons with the R *anova* function using BL-sur-cos and three partial models: one excluding the cosine similarity (BL-sur), and the other two excluding surprisal (BL-cos with BERT vectors and BL-cos with SGNS vectors), in order to check whether the two features make independent contributions. We obtained strongly significant *p*-values (*p* < 0.001) on both corpora, regardless of vector type and for all the eye-tracking features, indicating that both semantic relatedness and surprisal provide an independent and significant contribution.

Focusing now on BL-cos and BL-sur, the performance on **GECO** is reported in Tables 2, 3. BL-cos with BERT vectors: Delta cosine similarity is 585, Delta surprisal is 926 (surprisal: +341) (Table 3); BL-cos with SGNS vectors: Delta surprisal is 206, Delta cosine similarity is 696 (surprisal: −490) (Table 2); On **Provo** instead BL-cos with BERT vectors: Delta cosine similarity is 129, Delta surprisal is 324 (surprisal: +195) (Table 5); BL-cos with SGNS vectors: Delta surprisal is 112, Delta cosine is 158 (surprisal: −46) (Table 4). This first analysis shows that BL-cos and BL-sur have *quantitatively* similar behavior, suggesting that cosine and surprisal help to predict eye-tracking values to the same extent. A difference in the salience of the two features is instead highlighted by the Part-of-Speech analysis (see the related subsection below).

It is also clear that models using SGNS vectors have poorer performances than the ones relying on BERT. Not only, as already mentioned, the usage of BERT embeddings improves the performances of the BL-cos-sur model, but while comparing the BL-cos models and the BL-sur model, the first shows better performances than the latter only when BERT vectors are involved. This difference in the capability of BL-cos models in predicting eye-tracking features suggests that the findings in Frank (2017) might be influenced by the specific type of embedding model used for the experiments (SGNS).

Once confirmed that the model including both surprisal and cosine similarity is the one performing better, we performed further analysis focused on BL, BL-sur, and BL-cos only, in order to understand the individual contribution of the two computational metrics.

#### 4.1.3. Error analysis

In order to have a more fine-grained view of the performance differences between models BL-cos and BL-sur, we also analyzed the correlation between the Mean Absolute Error (MAE) of the models and word-level features. We tested the following features: target and previous word length, target and previous word frequency, target word length, target word position, fixation of the previous word (a boolean feature), and the reading complexity of the sentence from the beginning to the target word, which we computed using the Dale-Chall readability formula (Dale and Chall, 1948).

After we averaged the correlations among all the eye-tracking features to be predicted (see Appendix 2) we noticed that almost all the values are negative, suggesting that: (i) longer and more frequent words are easier to be predicted; (ii) words at the beginning of the sentence are harder to predict for our models, plausibly because a wider and richer context benefits both cosine similarity and surprisal; (iii) sentences with higher readability make better predictions possible. Even so, the correlations between MAE and these features are generally low, ranging from 0.002 for previous word length to 0.1 for target word length. However, it is possible to use these values for a comparison between models BL-cos and BL-sur. We notice that surprisal seems to be more sensitive to target word frequency and previous word fixation if compared to cosine similarity, while the latter shows slightly higher correlations with target word length and position within the sentence.

#### 4.1.4. POS analysis

Both GECO and Provo provide information regarding the part of speech (POS) of each word in the corpora. We used this information to check the performances of BL-cos and BL-sur on different POS. We first checked the average MAE of BL, BL-cos, and BL-sur for function words (pronouns, conjunctions, determiners, numeral, existential there's, prepositions, interjections) and content words (nouns, verbs, adverbs, adjectives) for each eye-tracking feature (Table 6). Then for a more detailed analysis, we ranked the words following the MAE values, and finally, we focused on the 10, 100, 500, and 1,000 words with the highest MAE.

**Table 6:** Average MAE on Provo and GECO content and function words from models BL, BL-cos, and BL-sur for the three eye-tracking features and their mean.

**Feature**	**Model**	**Word type**			
		**Content**		**Function**	
		**Provo**	**GECO**	**Provo**	**GECO**
TRT	BL	0.228	0.337	0.290	0.457
	BL-cos	0.217	0.333	0.281	0.457
	BL-sur	0.215	0.330	0.275	0.454
FFD	BL	0.180	0.295	0.246	0.425
	BL-cos	0.159	0.281	0.216	0.422
	BL-sur	0.172	0.289	0.236	0.423
NF	BL	0.178	0.228	0.147	0.187
	BL-cos	0.177	0.228	0.132	0.184
	BL-sur	0.170	0.226	0.140	0.185
Avg	BL	0.195	0.287	0.228	0.356
	BL-cos	**0.185**	**0.281**	**0.210**	0.354
	BL-sur	0.186	0.282	0.217	0.354

The bold formatting indicates the lowest MAE averaged over the 3 eye tracking features.

We found that for all three models function words are harder to be predicted than content words, especially coordinating conjunctions and pronouns. Noticeably, previous research had already found that the semantics of function words is difficult to model even for Transformers (Kim et al., 2019), and that fine-tuned multilingual Transformer model struggle the most with the prediction of their fixation metrics (Hollenstein et al., 2022b). Regarding the performances of BL-cos and BL-sur, even if both cosine similarity and surprisal help in lowering the average MAE, if compared to the baseline, cosine similarity employment improves slightly more the performance of the model for both content words and function words.

### 4.2. Eye-tracking features analysis

While comparing the different models, it was clear that some performance differences were due to the eye-tracking feature the models had to predict. For example, the data showed in the Avg column of Tables 2–5 are mean values computed using the AICc scores of TRT, FFD, and NF, but if we focus on the performances of models BL-cos and BL-sur, depending on the target eye-tracking features, we notice some interesting and substantial differences: on TRT cosine similarity-only and surprisal-only models follow the general tendency we described in Section 4.1 (i.e., surprisal better than cosine similarity when BL-cos makes use of SGNS vectors to compute cosine), but with cosine similarity performing generally slightly better than surprisal; on FFD the model using baseline regression features and cosine similarity only performs consistently better, except when using SGNS on GECO (but not on Provo), while on NF model BL-sur outperforms BL-cos on both corpora, even when using BERT vectors in BL-cos.

In the analysis of the correlations between models MAE and word features, we found that for TRT and FFD, the highest correlation (especially on GECO) is the one between MAE and the word length. Since it is a negative correlation, we can conclude that shorter words induce higher MAE: The shorter the word, the harder for the model to predict the feature value. On the other hand, with NF, word length has the highest, but *positive*, correlation with the MAE, thus suggesting that for this eye-tracking feature shorter words are easier to be predicted. Finally, for all the eye-tracking features on both corpora, word frequency is negatively correlated. As expected, prediction is more difficult for the rarest words.

When we checked the contribution of BL-cos and BL-sur in comparison to the baseline for different parts of speech, we noticed that for FFD cosine similarity generally decreases the MAE, while for TRT surprisal gives a generally higher contribution, except for verbs and adjectives (Tables 7, 8). Regarding NF, cosine similarity lowers the MAE for function words, while surprisal has a major impact on content words. However, for the NF feature content words are less easily predicted.

**Table 7:** Average MAE on Provo content words.

**Model**	**TRT**				**FFD**				**NF**			
	**N**	**RB**	**V**	**J**	**N**	**RB**	**V**	**J**	**N**	**RB**	**V**	**J**
BL	0.243	0.242	0.210	0.209	0.190	0.184	0.171	0.166	0.195	0.180	0.152	0.180
BL-cos	0.231	0.243	**0.198**	**0.199**	**0.178**	0.184	**0.157**	**0.154**	0.192	0.178	**0.149**	0.179
BL-sur	**0.228**	**0.221**	0.200	0.205	0.181	**0.176**	0.163	0.163	**0.183**	**0.171**	0.150	**0.178**

N, nouns; RB, adverbs; V, verbs; J, adjectives-for the three eye-tracking features. The bold formatting indicates the values with the lowest MAE of each POS within each eye-tracking feature.

**Table 8:** Average MAE on GECO content words.

**Model**	**TRT**				**FFD**				**NF**			
	**N**	**RB**	**V**	**J**	**N**	**RB**	**V**	**J**	**N**	**RB**	**V**	**J**
BL	0.335	0.365	0.334	0.309	0.289	0.322	0.294	0.273	0.238	0.226	0.217	0.242
BL-cos	0.328	0.367	0.332	0.301	0.280	0.323	0.292	0.264	0.237	0.226	0.217	0.241
BL-sur	**0.323**	**0.360**	0.332	**0.299**	0.280	**0.320**	0.292	**0.262**	**0.234**	**0.225**	**0.216**	**0.240**

N, nouns; RB, adverbs; V, verbs; J, adjectives-for the three eye-tracking features. The bold formatting indicates the values with the lowest MAE of each POS within each eye-tracking feature.

We surmise that the different performances of BL-sur and BL-cos in predicting these three eye-tracking features might be explained by taking into account the reading process stage each feature is related to. On one hand, since FFD is typically associated with early stages of reading, such as lexical information process, it is not surprising that the model relying on semantic relatedness between the context and the target word performs better. On the other hand, the performances of BL-cos and BL-sur on TRT and NF, features that reflect later stages of the reading process, including information-structural integration, may suggest that predictability is a key factor in handling syntagmatic relations and integrating semantic and syntactic information.

## 5. Conclusion

In this paper, we implemented four different kinds of regression models to predict three eye-tracking features of two corpora collecting eye movements data, with the aim of investigating the role and interplay between distributional measures of target-context semantic relatedness, and target surprisal, as computed with a state-of-the-art neural language model. The main research question was whether semantic relatedness is indeed made redundant by surprisal, as argued by Frank (2017), or instead plays an independent role in explaining eye-tracking data. The models include: (i) a baseline with word-level features, (ii) the same baseline with cosine similarity, (iii) the baseline with surprisal, iv) the baseline with both cosine similarity and surprisal.

Our results show that the complete model systematically outperforms the others for every eye-tracking feature and that both semantic relatedness and surprisal benefit the prediction of eye-tracking features, given the performance drop while factoring one of them out. Surprisal and distributional semantic relatedness clearly overlap, especially since the latter is nowadays commonly computed using word embeddings produced by DSMs trained with a prediction objective, like the one that surprisal formalizes. Yet, they capture different linguistic dimensions. Surprisal models the *syntagmatic* predictability of a word, given the preceding ones. On the other hand, both static and contextual DSMs use prediction as a distributional signal to form internal representations of lexical meaning that capture information more directly pertaining to the *paradigmatic* dimension, such as belonging to the same semantic classes and domains or sharing similar features. For instance, the words *pie* and *cake* are paradigmatically related because they share several salient attributes, such as being edible, sweet, etc. (Chersoni et al., 2021) showed that word embeddings encode a vast range of linguistically and cognitively relevant semantic features. Therefore, the results of our analyzes suggest that, despite their overlap, corpus-based semantic relatedness and surprisal capture different dimensions that play an autonomous role during reading. While surprisal reflects how predictable the target word is from the previous context, semantic relatedness models how coherent the meaning of the target is with respect to the context one (e.g., they belong to the same semantic field or describe a prototypical situation). Frank and Willems (2017) found that syntagmatic surprisal and paradigmatic semantic relatedness can have neurally distinguishable effects during language comprehension. Our analyzes show that their independent effect can be detected in eye-tracking data too.

We also analyzed whether the relatedness and surprisal have a differential effect depending on the target part-of-speech. Comparing the average MAE of our models, we noticed that surprisal mainly helps to improve the model's performances on content words, while the contribution of semantic relatedness includes function words as well. Finally, we investigated whether the interplay between surprisal and relatedness is affected by the type of word embeddings used to compute the latter, in particular considering the difference between static DSMs (SGNS) and contextual ones (BERT). The experiments show that when using BERT vectors, which are inherently able to account for context-dependent meaning shifts and carry out an implicit form of word-sense disambiguation, the model **BL-cos** performs better than **BL-sur**, while static vectors make the latter outrank the model using semantic relatedness only. Overall, our findings suggest that the kind of word embedding employed for computing vector distances has a significant impact, which may explain the differences from the findings by Frank (2017).

The present work admittedly has some limitations. For example, we employed and compared a restricted pool of language models and word embedding models, and a possible future direction could be testing other, more recent models (e.g., XLNet Yang et al. 2019, among others, RoBERTa Liu et al., 2019), or different static embedding models (e.g., GloVe Pennington et al., 2014, FastText Bojanowski et al., 2017). A particularly interesting issue, raised by some recent works, is the relationship between the size of a language model and its capacity to model human behavioral data (Oh and Schuler, 2022; Shain et al., 2022). In particular, Oh and Schuler (2022) found that larger language models are worse at predicting human reading times: larger models tend to be less surprised by open-class words because they have been trained on many more word sequences than those available to humans. Moreover, phenomena of inverse scaling have also been reported for language modeling of negations (Jang et al., 2022) and quantifiers (Kalouli et al., 2022; Michaelov and Bergen, 2022). It might be worth testing whether this increasing lack of alignment with human performance as scale increases can be observed also at the level of similarity estimation with the embeddings, or it is an effect limited to language model predictions. With this purpose, it could be interesting to compare embedding models of different size with BERT, and see if there are differences in modeling open class vs. function words.

Another limitation is due to the fact that we used English materials only, and this leaves open the question whether our results would apply to other languages. An interesting research path to pursue is to compare models with cosine similarity and surprisal using multilingual data. In fact, we plan to extend our analyzes to the recently-published MECO corpus (Siegelman et al., 2022), which provides eye-tracking data on comparable texts for 13 different languages.

Finally, if the importance and independence of surprisal and semantic relatedness are clear, given the results shown in the present paper, a preliminary feature importance analysis using a random forest regression model (see Appendix 3) revealed how target and previous word lengths are the features with the higher impact, and most importantly, surprisal systematically seems to have a larger effect on the model compared to cosine similarity. These preliminary results suggest one further possible research direction: the employment and comparison of different models and a consequent feature importance analysis, in order to find even more generalizable insights regarding the role of semantic relatedness and predictability in the reading process.

## Data availability statement

The original contributions presented in the study are included in the article/Supplementary material, further inquiries can be directed to the corresponding author.

## Author contributions

AL and EC contributed to the conception and design of the study. LS was responsible for the coding part, the data analysis, and the creation of the first draft of the manuscript. AL, EC, and LS contributed equally to the final form of the manuscript. All authors contributed to the article and approved the submitted version.

## Footnotes

^1^ https://expsy.ugent.be/downloads/geco/

^2^ https://osf.io/sjefs/

^3^The Wikipedia frequencies were extracted from https://github.com/IlyaSemenov/wikipedia-word-frequency
