# Correction: A study on surprisal and semantic relatedness for eye-tracking data prediction

**DOI:** 10.3389/fpsyg.2025.1749716

**Published:** 2025-12-17

**Authors:** Lavinia Salicchi, Emmanuele Chersoni, Alessandro Lenci

**Affiliations:** 1 Department of Chinese and Bilingual Studies, The Hong Kong Polytechnic University, Kowloon, Hong Kong SAR, China; 2 Computational Linguistics Laboratory (CoLing Lab), University of Pisa, Pisa, Italy

**Keywords:** cognitive modeling, surprisal, semantic relatedness, cosine similarity, language models, distributional semantics, eye-tracking

In the published article, there was a mistake in the corresponding author allocation.

Lavinia Salicchi was erroneously designated the corresponding author.

The correct correspondence appears below:

Emmanuele Chersoni, emmanuele.chersoni@polyu.edu.hk

The original version of this article has been updated.

