# Supplementary material for: A study on surprisal and semantic relatedness for eye-tracking data prediction
Source: Front Psychol. 2023 Feb 2;14:1112365. doi: 10.3389/fpsyg.2023.1112365 (PMC9931754; doi:10.3389/fpsyg.2023.1112365)
Supplement: Supplementary file 1 [file Data_Sheet_1.pdf]

Appendix

1 APPENDIX 1: SIGNIFICANCE SCORES - TOTAL READING TIME MODEL 4

Regression feature	P-value	
prev_len	0.000173	***
prev_freq	0.613098	
word_len	0.003388	**
word_freq	0.14597	
word_position	0.289876	
prev_fix	0.001364	**
sur_GPT	<2.00E-16	***
cos_BERT	<2.00E-16	***
prev_len:prev_freq	0.827092	
prev_len:word_len	0.004594	**
prev_freq:word_len	0.41442	
prev_len:word_freq	0.006895	**
prev_freq:word_freq	0.229562	
word_len:word_freq	0.506333	
prev_len:word_position	0.183116	
prev_freq:word_position	0.710103	
word_len:word_position	0.746884	
word_freq:word_position	0.525717	
prev_len:prev_fix	8.47E-05	***
prev_freq:prev_fix	0.616819	
word_len:prev_fix	0.113273	
word_freq:prev_fix	0.534924	
word_position:prev_fix	0.28867	
prev_len:prev_freq:word_len	0.593692	
prev_len:prev_freq:word_freq	0.335514	
prev_len:word_len:word_freq	0.034237	*
prev_freq:word_len:word_freq	0.242609	
prev_len:prev_freq:word_position	0.47928	
prev_len:word_len:word_position	0.582671	
prev_freq:word_len:word_position	0.902138	
prev_len:word_freq:word_position	0.334066	
prev_freq:word_freq:word_position	0.888465	
word_len:word_freq:word_position	0.976447	
prev_len:prev_freq:prev_fix	0.828081	
prev_len:word_len:prev_fix	0.003521	**
prev_freq:word_len:prev_fix	0.414459	
prev_len:word_freq:prev_fix	0.006125	**
prev_freq:word_freq:prev_fix	0.231859	
word_len:word_freq:prev_fix	0.810387	
prev_len:word_position:prev_fix	0.17894	
prev_freq:word_position:prev_fix	0.709546	
word_len:word_position:prev_fix	0.749998	
word_freq:word_position:prev_fix	0.545097	
prev_len:prev_freq:word_len:word_freq	0.34081	
prev_len:prev_freq:word_len:word_position	0.85981	
prev_len:prev_freq:word_freq:word_position	0.85923	
prev_len:word_len:word_freq:word_position	0.84234	
prev_freq:word_len:word_freq:word_position	0.574659	
prev_len:prev_freq:word_len:prev_fix	0.596571	
prev_len:prev_freq:word_freq:prev_fix	0.340557	
prev_len:word_len:word_freq:prev_fix	0.033951	*
prev_freq:word_len:word_freq:prev_fix	0.245644	
prev_len:prev_freq:word_position:prev_fix	0.476755	
prev_len:word_len:word_position:prev_fix	0.5783	
prev_freq:word_len:word_position:prev_fix	0.898595	
prev_len:word_freq:word_position:prev_fix	0.328633	
prev_freq:word_freq:word_position:prev_fix	0.885179	
word_len:word_freq:word_position:prev_fix	0.965692	
prev_len:prev_freq:word_len:word_freq:word_position	0.780146	
prev_len:prev_freq:word_len:word_freq:prev_fix	0.345232	
prev_len:prev_freq:word_len:word_position:prev_fix	0.858484	
prev_len:prev_freq:word_freq:word_position:prev_fix	0.854513	
prev_len:word_len:word_freq:word_position:prev_fix	0.838069	
prev_freq:word_len:word_freq:word_position:prev_fix	0.572249	
prev_len:prev_freq:word_len:word_freq:word_position:prev_fix	0.782975	

Table S1. BL-sur-cos GECO BERT

Regression feature	P-value	
prev_len	0.000699	***
prev_freq	0.552414	
word_len	0.002424	**
word_freq	1.32E-01	
word_position	3.43E-01	
prev_fix	4.09E-03	**
sur_GPT	<2.00E-16	***
cos_SGNS	<2.00E-16	***
prev_len:prev_freq	0.744339	
prev_len:word_len	0.0066	**
prev_freq:word_len	0.412876	
prev_len:word_freq	0.005771	**
prev_freq:word_freq	0.206059	
word_len:word_freq	0.402396	
prev_len:word_position	0.266802	
prev_freq:word_position	0.685429	
word_len:word_position	0.651723	
word_freq:word_position	0.466753	
prev_len:prev_fix	0.000353	***
prev_freq:prev_fix	0.564002	
word_len:prev_fix	0.083224	.
word_freq:prev_fix	0.346399	
word_position:prev_fix	0.350155	
prev_len:prev_freq:word_len	0.56283	
prev_len:prev_freq:word_freq	0.302194	
prev_len:word_len:word_freq	0.032301	*
prev_freq:word_len:word_freq	0.235093	
prev_len:prev_freq:word_position	0.515943	
prev_len:word_len:word_position	0.548879	
prev_freq:word_len:word_position	0.992679	
prev_len:word_freq:word_position	0.343154	
prev_freq:word_freq:word_position	0.942817	
word_len:word_freq:word_position	0.873858	
prev_len:prev_freq:prev_fix	0.747194	
prev_len:word_len:prev_fix	0.005061	**
prev_freq:word_len:prev_fix	0.417455	
prev_len:word_freq:prev_fix	0.005205	**
prev_freq:word_freq:prev_fix	0.210133	
word_len:word_freq:prev_fix	0.601061	
prev_len:word_position:prev_fix	0.262443	
prev_freq:word_position:prev_fix	0.688897	
word_len:word_position:prev_fix	0.664249	
word_freq:word_position:prev_fix	0.497757	
prev_len:prev_freq:word_len:word_freq	0.316689	
prev_len:prev_freq:word_len:word_position	0.791486	
prev_len:prev_freq:word_freq:word_position	0.836863	
prev_len:word_len:word_freq:word_position	0.747324	
prev_freq:word_len:word_freq:word_position	0.647865	
prev_len:prev_freq:word_len:prev_fix	0.566546	
prev_len:prev_freq:word_freq:prev_fix	0.307375	
prev_len:word_len:word_freq:prev_fix	0.032126	*
prev_freq:word_len:word_freq:prev_fix	0.239071	
prev_len:prev_freq:word_position:prev_fix	0.514031	
prev_len:word_len:word_position:prev_fix	0.545749	
prev_freq:word_len:word_position:prev_fix	0.998868	
prev_len:word_freq:word_position:prev_fix	0.338672	
prev_freq:word_freq:word_position:prev_fix	0.935831	
word_len:word_freq:word_position:prev_fix	0.894302	
prev_len:prev_freq:word_len:word_freq:word_position	0.856575	
prev_len:prev_freq:word_len:word_freq:prev_fix	0.321318	
prev_len:prev_freq:word_len:word_position:prev_fix	0.790865	
prev_len:prev_freq:word_freq:word_position:prev_fix	0.832381	
prev_len:word_len:word_freq:word_position:prev_fix	0.743896	
prev_freq:word_len:word_freq:word_position:prev_fix	0.642252	
prev_len:prev_freq:word_len:word_freq:word_position:prev_fix	0.859469	

Table S2. BL-sur-cos GECO SGNS

Regression feature	P-value	
prev_len	0.28641	
prev_freq	0.30011	
word_len	2.38E-07	***
word_freq	4.37E-01	
word_position	2.71E-01	
prev_fix	0.00237	**
sur_GPT	<2E-16	***
cos_BERT	<2E-16	***
prev_len:prev_freq	0.85958	
prev_len:word_len	0.2353	
prev_freq:word_len	0.22659	
prev_len:word_freq	0.77062	
prev_freq:word_freq	0.209	
word_len:word_freq	0.97701	
prev_len:word_position	0.21162	
prev_freq:word_position	0.31084	
word_len:word_position	0.18996	
word_freq:word_position	0.4577	
prev_len:prev_fix	-	
prev_freq:prev_fix	-	
word_len:prev_fix	0.02915	*
word_freq:prev_fix	0.4721	
word_position:prev_fix	-	
prev_len:prev_freq:word_len	0.41355	
prev_len:prev_freq:word_freq	0.799	
prev_len:word_len:word_freq	0.74179	
prev_freq:word_len:word_freq	0.25133	
prev_len:prev_freq:word_position	0.37566	
prev_len:word_len:word_position	0.13789	
prev_freq:word_len:word_position	0.26883	
prev_len:word_freq:word_position	0.40755	
prev_freq:word_freq:word_position	0.47922	
word_len:word_freq:word_position	0.3823	
prev_len:prev_freq:prev_fix	-	
prev_len:word_len:prev_fix	-	
prev_freq:word_len:prev_fix	-	
prev_len:word_freq:prev_fix	-	
prev_freq:word_freq:prev_fix	-	
word_len:word_freq:prev_fix	0.51518	
prev_len:word_position:prev_fix	-	
prev_freq:word_position:prev_fix	-	
word_len:word_position:prev_fix	-	
word_freq:word_position:prev_fix	-	
prev_len:prev_freq:word_len:word_freq	0.62329	
prev_len:prev_freq:word_len:word_position	0.31611	
prev_len:prev_freq:word_freq:word_position	0.6144	
prev_len:word_len:word_freq:word_position	0.36677	
prev_freq:word_len:word_freq:word_position	0.44175	
prev_len:prev_freq:word_len:prev_fix	-	
prev_len:prev_freq:word_freq:prev_fix	-	
prev_len:word_len:word_freq:prev_fix	-	
prev_freq:word_len:word_freq:prev_fix	-	
prev_len:prev_freq:word_position:prev_fix	-	
prev_len:word_len:word_position:prev_fix	-	
prev_freq:word_len:word_position:prev_fix	-	
prev_len:word_freq:word_position:prev_fix	-	
prev_freq:word_freq:word_position:prev_fix	-	
word_len:word_freq:word_position:prev_fix	-	
prev_len:prev_freq:word_len:word_freq:word_position	0.58284	
prev_len:prev_freq:word_len:word_freq:prev_fix	-	
prev_len:prev_freq:word_len:word_position:prev_fix	-	
prev_len:prev_freq:word_freq:word_position:prev_fix	-	
prev_len:word_len:word_freq:word_position:prev_fix	-	
prev_freq:word_len:word_freq:word_position:prev_fix	-	
prev_len:prev_freq:word_len:word_freq:word_position:prev_fix	-	

Table S3. BL-sur-cos Provo BERT

Regression feature	P-value	
prev_len	0.18607	
prev_freq	0.18038	
word_len	5.10E-07	***
word_freq	0.46545	
word_position	2.30E-01	
prev_fix	1.51E-03	**
sur_GPT	<2.00E-16	***
cos_SGNS	<2.00E-16	***
prev_len:prev_freq	8.66E-01	
prev_len:word_len	0.0846	.
prev_freq:word_len	0.13109	
prev_len:word_freq	0.69703	
prev_freq:word_freq	0.14042	
word_len:word_freq	0.91238	
prev_len:word_position	0.09704	.
prev_freq:word_position	0.28707	
word_len:word_position	0.18239	
word_freq:word_position	0.45885	
prev_len:prev_fix	-	
prev_freq:prev_fix	-	
word_len:prev_fix	0.00871	**
word_freq:prev_fix	0.20894	
word_position:prev_fix	-	
prev_len:prev_freq:word_len	0.26654	
prev_len:prev_freq:word_freq	0.85531	
prev_len:word_len:word_freq	0.42944	
prev_freq:word_len:word_freq	0.16339	
prev_len:prev_freq:word_position	0.24598	
prev_len:word_len:word_position	0.08612	.
prev_freq:word_len:word_position	0.29391	
prev_len:word_freq:word_position	0.29565	
prev_freq:word_freq:word_position	0.50804	
word_len:word_freq:word_position	0.43144	
prev_len:prev_freq:prev_fix	-	
prev_len:word_len:prev_fix	-	
prev_freq:word_len:prev_fix	-	
prev_len:word_freq:prev_fix	-	
prev_freq:word_freq:prev_fix	-	
word_len:word_freq:prev_fix	0.23868	
prev_len:word_position:prev_fix	-	
prev_freq:word_position:prev_fix	-	
word_len:word_position:prev_fix	-	
word_freq:word_position:prev_fix	-	
prev_len:prev_freq:word_len:word_freq	0.42096	
prev_len:prev_freq:word_len:word_position	0.29149	
prev_len:prev_freq:word_freq:word_position	0.53869	
prev_len:word_len:word_freq:word_position	0.33627	
prev_freq:word_len:word_freq:word_position	0.51261	
prev_len:prev_freq:word_len:prev_fix	-	
prev_len:prev_freq:word_freq:prev_fix	-	
prev_len:word_len:word_freq:prev_fix	-	
prev_freq:word_len:word_freq:prev_fix	-	
prev_len:prev_freq:word_position:prev_fix	-	
prev_len:word_len:word_position:prev_fix	-	
prev_freq:word_len:word_position:prev_fix	-	
prev_len:word_freq:word_position:prev_fix	-	
prev_freq:word_freq:word_position:prev_fix	-	
word_len:word_freq:word_position:prev_fix	-	
prev_len:prev_freq:word_len:word_freq:word_position	0.61993	
prev_len:prev_freq:word_len:word_freq:prev_fix	-	
prev_len:prev_freq:word_len:word_position:prev_fix	-	
prev_len:prev_freq:word_freq:word_position:prev_fix	-	
prev_len:word_len:word_freq:word_position:prev_fix	-	
prev_freq:word_len:word_freq:word_position:prev_fix	-	
prev_len:prev_freq:word_len:word_freq:word_position:prev_fix	-	

Table S4. BL-sur-cos Provo SGNS

2 APPENDIX 3: CORRELATIONS BETWEEN MEAN ABSOLUTE ERROR AND WORD FEATURES.

Feature	Model	GECO			Provo			Avg
		TRT	FFD	NF	TRT	FFD	NF	
**prev_len**								
	**BL**	0.0026	0.0151	-0.0891	0.0714	0.1154	-0.0484	0.0112
	**BL+cos**	0.0008	0.0141	-0.0910	0.0572	0.0960	-0.0602	0.0028
	**BL+sur**	0.0018	0.0153	-0.0905	0.0671	0.1078	-0.0640	0.0063
**prev_freq**								
	**BL**	-0.0593	-0.0557	-0.0483	-0.0777	-0.0930	-0.0211	-0.0592
	**BL+cos**	-0.0655	-0.0644	-0.0501	-0.0886	-0.1135	-0.0178	-0.0666
	**BL+sur**	-0.0683	-0.0626	-0.0525	-0.0789	-0.1098	-0.0268	-0.0665
**word_len**								
	**BL**	-0.1957	-0.2434	0.1426	-0.1522	-0.2071	0.1360	-0.0866
	**BL+cos**	-0.1997	-0.2475	0.1439	-0.1527	-0.2062	0.1616	-0.0834
	**BL+sur**	-0.2025	-0.2491	0.1440	-0.1419	-0.1859	0.1473	-0.0814
**word_freq**								
	**BL**	-0.0040	0.0069	-0.1261	0.0686	0.0938	-0.1284	-0.0149
	**BL+cos**	0.0029	0.0156	-0.1242	0.0903	0.1188	-0.1284	-0.0042
	**BL+sur**	0.0043	0.0177	-0.1221	0.0703	0.0901	-0.1206	-0.0101
**word_position**								
	**BL**	-0.0566	-0.0514	-0.0674	-0.0235	-0.0124	-0.0598	-0.0452
	**BL+cos**	-0.0596	-0.0558	-0.0704	-0.0149	-0.0090	-0.0652	-0.0458
	**BL+sur**	-0.0567	-0.0514	-0.0703	-0.0023	0.0049	-0.0422	-0.0363
**prev_fix**								
	**BL**	-0.0988	-0.1076	-0.0681	-0.0765	-0.0706	-0.1072	-0.0881
	**BL+cos**	-0.1011	-0.1105	-0.0695	-0.0851	-0.0837	-0.1122	-0.0937
	**BL+sur**	-0.1006	-0.1091	-0.0723	-0.0872	-0.0780	-0.1197	-0.0945
**Dale_Chall**								
	**BL**	-0.0217	-0.0288	0.0122	0.0030	0.0184	0.0268	0.0017
	**BL+cos**	-0.0238	-0.0303	0.0114	-0.0257	-0.0075	0.0115	-0.0107
	**BL+sur**	-0.0209	-0.0285	0.0118	0.0152	0.0291	0.0186	0.0042

Table S5. Correlations between Average MEA and baseline features, for each eye-tracking feature.

3 APPENDIX 3: RANDOM FOREST - FEATURE IMPORTANCE ANALYSIS

Provo (BERT)

	TRT		FFD		NF	
	IncMSE	IncNodePurity	IncMSE	IncNodePurity	IncMSE	IncNodePurity
**prev_len**	43.01	38.59	48.63	27.79	27.03	17.66
**prev_freq**	29.50	41.01	29.64	26.59	20.22	20.73
**word_len**	64.90	303.06	67.80	176.26	66.55	205.37
**word_freq**	31.25	185.96	32.53	99.33	29.14	118.22
**word_position**	22.83	30.26	17.94	16.68	19.49	18.78
**prev_fix**	9.50	2.25	10.87	1.74	8.13	1.59
**sur_GPT**	**37.47**	70.40	**34.98**	40.53	**29.12**	48.89
**cos_BERT**	28.15	155.89	27.12	105.60	28.40	82.35

Provo (SGNS)

	TRT		FFD		NF	
	IncMSE	IncNodePurity	IncMSE	IncNodePurity	IncMSE	IncNodePurity
**prev_len**	41.28	39.60	44.66	28.13	27.61	17.57
**prev_freq**	29.73	43.63	32.36	28.35	19.40	21.24
**word_len**	71.63	322.88	78.61	199.07	74.52	218.77
**word_freq**	35.31	206.74	35.99	119.34	33.74	126.72
**word_position**	24.52	31.47	19.94	17.98	23.23	19.49
**prev_fix**	9.64	1.57	11.40	1.64	6.06	1.17
**sur_GPT**	**35.09**	80.16	**33.99**	42.29	**29.06**	47.62
**cos_SGNS**	30.40	106.00	30.02	57.39	27.28	62.45

GECO (BERT)

	TRT		FFD		NF	
	IncMSE	IncNodePurity	IncMSE	IncNodePurity	IncMSE	IncNodePurity
**prev_len**	29.86	1,091.45	20.95	898.75	37.60	300.64
**prev_freq**	45.31	1,380.54	24.22	1,119.14	25.00	388.21
**word_len**	103.52	8,362.78	95.47	6,027.04	106.63	3,539.48
**word_freq**	38.97	3,099.18	37.65	2,122.29	49.70	1,256.05
**word_position**	24.57	1,234.85	19.04	985.05	29.05	389.64
**prev_fix**	6.65	72.80	8.19	46.55	13.07	19.38
**sur_GPT**	**53.87**	2,821.75	**51.28**	2,150.31	**60.09**	956.37
**cos_BERT**	27.22	3,604.02	24.04	2,721.35	52.64	1,232.35

GECO (SGNS)

	TRT		FFD		NF	
	IncMSE	IncNodePurity	IncMSE	IncNodePurity	IncMSE	IncNodePurity
**prev_len**	32.07	1,167.53	28.22	954.31	29.54	310.97
**prev_freq**	50.24	1,406.12	47.87	1,134.57	46.95	393.88
**word_len**	107.65	8,908.50	101.12	6,078.00	120.84	3,625.97
**word_freq**	45.82	3,435.67	44.53	2,687.90	48.52	1,512.98
**word_position**	23.90	1,276.70	31.35	1,022.79	22.76	400.27
**prev_fix**	10.60	54.20	10.71	52.41	12.03	20.37
**sur_GPT**	**58.20**	3,098.06	**52.95**	2,265.95	**60.40**	1,028.44
**cos_SGNS**	39.28	2,325.80	39.61	1,808.41	37.91	796.95

Table S6. Random forest feature importance analysis for each corpus and eye-tracking feature, using SGNS or BERT vectors for the semantic relatedness model feature.
